# Characterization of Flavonoid Glycosides from Fenugreek (*Trigonella foenum-graecum*) Crude Seeds by HPLC–DAD–ESI/MS Analysis

**DOI:** 10.3390/ijms151120668

**Published:** 2014-11-11

**Authors:** Zakia Benayad, Carmen Gómez-Cordovés, Nour Eddine Es-Safi

**Affiliations:** 1 Institute of Food science, Technology and Nutrition of the Spanish National Research Council (ICTAN–CSIC), Juan de la Cierva 3, Madrid 28006, Spain; E-Mail: cgcordoves@ifi.csic.es; 2 Team of Organic Chemistry and Physico-Chemical Studies, Ecole Normale Superieure, Mohammed V University of Rabat, P.O. Box 5118, 10200 Rabat, Morocco

**Keywords:** *Trigonella foenum-graecum*, fenugreek seeds, Accelerated Solvent Extractor (ASE), flavonoids, flavones, flavonols, acylated glycosides, hydrocinnamic acids, mass spectrometry, LC–MS analysis

## Abstract

Fenugreek (*Trigonella foenum-graecum*) is a medicinal plant which is widely used for its pharmacological properties. In this study the phenolic composition of fenugreek crude seeds originating from Morocco has been investigated. Extraction was performed from defatted seeds by a hydromethanolic solution using an Accelerated Solvent Extractor. HPLC technique coupled to negative ion electrospray ionization mass spectrometry and diode array detection was employed to identify the polyphenol in the obtained extract. The obtained results allowed the detection of 32 phenolic compounds among which various flavonoid glycosides and phenolic acids have been tentatively identified on the basis of their UV and MS spectra, and comparisons with standards when available, as well as with literature data. A systematic study of the obtained MS spectra and the observed fragmentation showed that most of the identified compounds were acylated and non-acylated flavonoids with apigenin, luteolin and kaempferol as aglycons. Hydroxycinnamic acids mostly dominated by caffeic acid derivatives were also detected. The quantitative analysis of the identified compounds showed that the phenolic composition of the studied crude fenugreek seeds was predominantly acylated and non-acylated flavone derivatives with apigenin as the main aglycon.

## 1. Introduction

Fenugreek (*Trigonella foenum-graecum*) is an annual plant of the Fabaceae family. The seeds of this plant are used by people in Asia, Africa and Mediterranean countries as one of the ingredients in daily diets [1]. It is used in many domains including medicine, nutrition, beverages, fragrances, cosmetics and for other industrial purposes [2]. Fenugreek is known to have several pharmacological effects including hypoglycaemia [3,4], hypocholesterolemia [5,6], gastroprotective [7], chemopreventive [8], antioxidant [9,10], antiinflammatory, antipyretic [11] and appetite stimulation [12].

Regarding its phytochemical composition, previously reported data on fenugreek highlighted the presence of alkaloids [13], flavonoids and phenolic acids [13,14,15,16,17,18], polysaccharides [13], triterpenoids [19], steroidal sapogenins [20,21,22] and nicotinic acid [23].

In Morocco, fenugreek has been widely used as a spice crop for a long time. It is also widely used in traditional medicine as a tonic, as a remedy against stomach disorders, diabetes, fever, anaemia, constipation, and as a galactogogue and for stimulation of appetite [24]. In an ongoing program aiming at the study of bioactive natural products, we investigated the chemical composition of crude fenugreek seeds. The beneficial health effects of this plant prompted us to explore its secondary metabolites in order to provide major information about its chemical content. The increasing interest of nutritional and pharmacological power of different parts of the plant motivated this investigation. The objectives of this research work were the extraction of phenolic compounds from crude fenugreek (*Trigonella foenum-graecum*) seeds by Accelerated Solvent Extractor (ASE) and the subsequent characterization of the flavonoid glycosides through HPLC/DAD/MS/ESI analysis. This was achieved through acquisition of the product ion mass spectra of deprotonated phenolic glycosides and to explore the observed fragmentations. The presence of many phenolic compounds pertaining to acylated and non-acylated flavonoids in addition to phenolic acids was observed. Their structures were elucidated based on the obtained UV and MS spectra. Quantitative analysis of the individual identified phenolic compounds is also reported. This work presents thus a more complete description of the phenolic compounds present in the crude fenugreek seeds.

## 2. Results and Discussion

**Figure 1:**
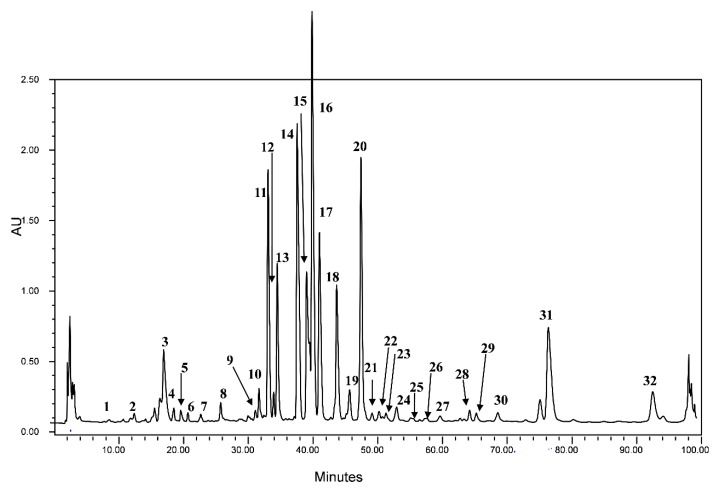
HPLC chromatogram of the studied fenugreek crude seeds recorded at 280 nm.

The extracted crude fenugreek seeds sample was analyzed by HPLC coupled to both diode array and mass spectrometry detectors. The latter was used with an electrospray ionization source in negative ion mode. ESI LC-MS analysis was performed using cone voltage providing useful additional fragmentation data. A representative UV chromatogram is shown in Figure 1 indicating that the used HPLC conditions allowed a good separation of a large percentage of compounds.

The HPLC separation profile revealed the presence of various chromatographic peaks in the studied sample extract. The structures of the separated compounds representing the major detected peaks and summarizing the obtained data for each of the detected chromatographic peak are discussed below. Compounds identification was based on the use of standard when available or on retention data, UV absorption, and ESI mass spectra and by comparison with literature data [25,26,27,28,29,30,31,32,33,34,35,36,37,38].

Online UV–visible spectra of the observed phenolic compounds were typical of flavone and flavonol glycosides in addition to phenolic acids. Most of the detected compounds showed UV absorptions maxima with two bands at 232–270 and 316–346 nm which are characteristic of flavonoids [39]. Some peaks with characteristic UV absorptions band for hydroxycinnamic acids were also detected.

The identification of the detected compounds was based on the search of the main molecular ions and also on some of the useful observed fragmentations. ESI LC–MS was performed using cone voltages with the higher cone voltage providing additional fragmentation data. Valuable information was also obtained concerning the presence and the nature of acyl groups. Compounds listed in Table 1 were restricted to those in which [M − H]^−^ ions were clearly detected. Among the observed peaks, some were too small to allow structural analyses.

Different compounds pertaining to flavonoids with *C*- and/or *O*-hexosides and/or pentosides and with or without acyl groups have been detected. According to the obtained data, and taking into account the previously reported results on flavonoids mass spectrometry fragmentations, the position of glycosylation was in 7-hydroxyl group for flavones and flavonols and 6C/8C position for *C*-glycosides [32,40,41,42]. Moreover, the determination of linkage type of the *O*- and/or *C-*glycosides and differentiation of the positional flavones and flavonols glycoside isomers was determined through comparison of the relative intensity of the product ions spectra [34,43,44,45]. On the other hand, the interglycosidic linkage involved between the sugar moieties in the *O*- and/or *C*-glycosyl flavones and flavonols was in 1→2 and 1→6 linkages [45,46].

**Table 1:** Chromatographic, spectral data and identification of non-acylated flavonoid glycosides in fenugreek crude seeds.

Peak	*R*_t_ (min)	λ_max_ (nm)	[M − H]^−^	Fragment Signals (*m*/*z*)	Compound Identification
9	31.74	234, 272, 334	593	473, 383, 353	apigenin 6,8-di *C*-hexoside (vicenin 2 isomer)
11	33.65	232, 272, 334	593	473, 383, 353	apigenin 6,8-di *C*-glucoside (vicenin 2)
13	35.06	232, 270, 336	593	473, 383, 353	apigenin 6,8-di *C*-hexoside (vicenin 2 isomer)
14	38.12	232, 270, 336	563	443, 383, 353	apigenin 8- *C*-xyloside-6-*C*-glucoside (vicenin 3)
17	41.53	230, 270, 336	563	443, 383, 353	apigenin 6- *C*-xyloside-8-*C*-glucoside (vicenin 1)
19	46.17	232, 272, 338	577	503, 473, 383, 353	apigenin 8- *C*-rhamnoside-6-*C*-glucoside
31	77.08	232, 270, 316	593	447, 429, 309, 285	kaempferol 7- *O*-rhamnosyl-(1→2)-glucoside

**Scheme 1:**
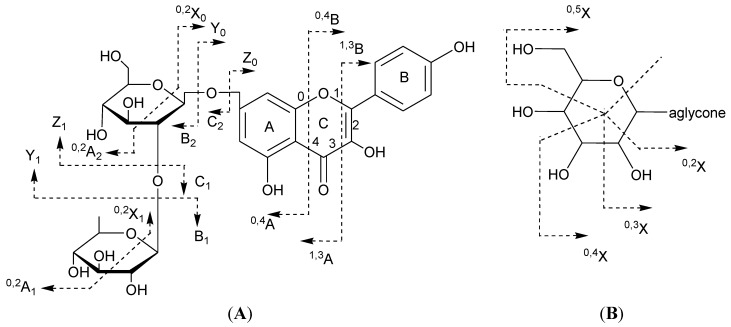
Ion nomenclature used for flavonoid glycosides (illustrated on kaempferol 7-*O*-rhamnosyl-(1→2)-glucoside (**A**) and a *C*-hexoside derivative (**B**)).

In flavonoids mass spectrometry, the major diagnostic fragmentations for flavonoid identification are those involving the cleavage of two C–C bonds of the C-ring giving two structurally informative fragment ions. These ions provide information on the number and type of substituents in A- and B-rings. In this paper, these fragment ions are designated according to the nomenclature previously proposed [47]. For free aglycone, the ^i,j^A and ^i,j^B labels refer to the fragments containing intact A- and B-rings, respectively, in which the superscripts *i* and *j* indicate the C-ring bonds that have been broken. For flavonoid glycosides, the classical nomenclature proposed by Domon and Costello for glycoconjugates was adopted to designate the fragmentations [33]: *^k^*^,*l*^X*_j_*, Y*^n^_j_*, Z*^n^_j_* represents the ions still containing the aglycone, where *j* is the number of the interglycosidic bonds broken (counted from the aglycon) and *k* and *l* denote the cleavage within the carbohydrate rings, *n* represents the position where the oligosaccharide is attached to the aglycone (Scheme 1).

The detected flavonoids were divided into non acylated and acylated glycosides. The first group consisted of flavone di-*C*-glycosides and flavonol *O*-diglycosides, which were characterized by mass losses corresponding to the fragmentations ^0,2^X_0_, ^0,3^X_0_, ^0,1^X_0_, ^1,5^X_0_ and −Y_j_^7^Y_0_^7^ of the sugar moieties. The second group consisted of flavone *O*,*C*-glycosides with an acyl moiety on the *C*-glycosyl due to the presence of losses corresponding to the fragmentations −Z_j_^−^ (acyl − 18), ^0,2^X_0_, ^0,3^X_0_ [35,36,45]. The acyl groups were dihydroferulic, hydroxyferulic, gallic, dihydrogallic, methoxygallic, quinic, malonyl and acetyl moieties with a predominance of the dihydrogallic acid.

### 2.1. Non-Acylated Flavone and Flavonol Glycosides

Compounds **9**, **11** and **13** present the same molecular ion at *m*/*z*: 593 [M − H]^−^ which was observed as base peak (Table 1). The MS data showed fragment ions at *m*/*z*: 383 (Ag + 113) and *m*/*z*: 353 (Ag + 83) which are characteristics of di *C*-glycosylflavone fragmentations [48]. Another fragment ion signal was observed at *m*/*z*: 473 [(M − H) − 120]^−^ and corresponding to the ^0,2''^X_0_. This signal was observed with a low relative intensity, indicating to the loss of another glycosyl residue [29,30]. A comparison of the relative intensity of the observed fragment signals showed that the signal located at *m*/*z* 383 was higher than that at *m*/*z* 473 suggesting an apigenin aglycone. Consequently these compounds were concluded to be apigenin 6,8-di *C*-hexoside isomers as observed in our previous spectral analysis of compounds from fenugreek germinated seeds [32]. Compound **11** presents fragmentations similar to those indicated for apigenin 6,8-di *C*-glucoside [16,49]. The structure of vicenin-2 was then proposed for this compound.

Compounds **14** and **17** present a molecular ion signal at *m*/*z*: 563 [M − H]^−^ (base peak). Additional fragment signals were observed at *m*/*z*: 473 [(M – H) − 90]^−^ (−^0,3''^X_0_), *m*/*z*: 443 [(M – H) − 120]^−^(−^0.2''^X_0_), *m*/*z*: 383 [(M – H) − 120 − 60]^−^ (−^0,3'''^X_0_) and *m*/*z*: 353 [(M – H) − 120 − 90 ]^−^ (−^0,2'''^X_0_). These neutral losses are characteristics of a glucosyl and pentosyl residues as previously reported [32,35,45]. The fragments ion signals observed at *m*/*z*: 383 (Ag + 113) and *m*/*z*: 353 (Ag + 83) are typical of di *C*-glycosylflavones as mentioned above. These results suggested thus that compounds **14** and **17** were isomers with skeletons consisting of apigenin with a pentosyl and a glucosyl linked to the 6 and 8 positions.

Taking into account the previously reported data concerning the potential of MS methods in the determination of the type of the substitution of a sugar at the 6 and 8 positions of a 6,8-di *C*-glycosylflavones where the preferential fragmentation of the sugar moiety at the 6 position has been shown and with regard to the obtained results, compound **17** was proposed to be apigenin 6-*C*-xyloside-8-*C*-glucoside (vicenin 1), while compound **14** was proposed to be apigenin 8-*C*-xyloside-6-*C*-glucoside (vicenin 3). This is in agreement with the results previously reported on fenugreek seeds [14,16,32].

Compound **19** presents a molecular ion signal at *m*/*z*: 577 [M − H]^−^. The observed fragmentation pattern was typical of a di *C*-glycosylflavone [48]. The MS data showed fragment ion signals at *m*/*z*: 503 [(M − H) − 74]^−^ (−^0,3''^X_0_) and *m*/*z*: 473 [(M − H) − 104]^−^ (−^0,2''^X_0_), which are characteristics of a *C*-linked rhamonsyl [35,37]. Other fragments signals at *m*/*z*: 383 [(M − H) − 90]^−^ (−^0,3'''^X_0_) and *m*/*z*: 353 [(M − H) − 120]^−^ (−^0,2'''^X_0_) were also observed and corresponded to the loss of a *C*-glucosyl moiety [36,45]. The observed relative intensity of the fragment ion signals released from the *C*-glucosyl moiety were higher than those of the *C*-rhamonsyl one in agreement with the glucosyl moiety fixed at the 6 position while the the rhamnosyl group was linked at the 8 position [42]. Therefore compound **19** was concluded to be apigenin 8-*C*-rhamnosyl 6-*C*-glucoside.

Compound **31** presents a molecular ion at *m*/*z*: 593 [M − H]^−^. The obtained MS data also showed fragment ion signals at *m*/*z*: 447 [(M − H) −146]^−^ (−Y_1'''_^7^), *m*/*z*: 327 [(M − H) − 146 − 120]^−^ (−^0,2''^X_0_) and *m*/*z*: 285 [(M − H) − 146 − 162]^−^ (−Y_0_^7^). The latter signal corresponding to the aglycon moiety indicated a loss of the *O*-rhamanosyglucosyl in agreement with previously reported data [31]. According to Cuyckens *et al.* (2000) [46], the presence of the ion (−^0,2''^X_0_) with a high abundance is characteristic of the 1→2 isomer. Therefore, this compound could be kaempferol 7-*O*-(2''-rhamnosyl)-glucoside.

### 2.2. Acylated Flavone and Flavonol Glycosides

Compound **10** presents a molecular ion at *m*/*z*: 771 [M − H]^−^ (Table 2). The MS data showed fragment signals at *m*/*z*: 383 (Ag + 113) (−^0,3'''^X) and *m*/*z*: 353 (Ag + 83) (−^0,2'''^X_0_) in agreement with a di *C*-glycosylflavone [48]. Additional fragment signals were observed at *m*/*z*: 593 [M − H − 178]^−^(Y^2''^) (acyl − 18) and *m*/*z*: 473 [(M − H) − 178 − 120]^−^ (−^0.2''^X_0_) suggesting an *O*-(Acyl)-glycosylflavone. The observed loss of 178 amu is in agreement with the molecular mass of a dehydrated dihydroferulic acid moiety connected through an interglycosidic 1→2 linkage to the *C*-glucosyl unit. This is due to the high relative intensity of the signal observed at *m*/*z*: 593 which was observed as base peak [43,45]. From these results, compound **10** was tentatively concluded to be apigenin 6-*C*-glucosyl 8-*C*-(2''-*O*-dihydroferuloyl)-glucoside.

The compound **12** showed a mass molecular ion at *m*/*z*: 749 [M − H]^−^. The MS data also showed a fragment signal at *m*/*z*: 593 [M − H − 156]^−^ corresponding to the loss of a rhamnosyl moiety. Further ion signals were observed at *m*/*z*: 503 [(M − H) − 156 − 90]^−^ (^0,3^X_0_^−^), 473 [(M − H) − 156 − 120]^−^ (^0,2^X_0_^−^), with a low relative intensity, indicating the loss of a glucose residue. In the same spectrum a fragment ions signals were observed at *m*/*z*: 383 [(M − H) − 156 − 120 − 90]^−^ and 353 [(M − H) − 156 − 120 − 120]^−^, with high relative intensities, indicating to the loss of another glucose residue. Moreover, the ion fragment signals observed at *m*/*z*: 383 (aglycone + 113) and *m*/*z*: 353 (aglycone + 83), are characteristic of a di *C*-glycosylflavone fragmentation [48] with apigenin as aglycone. The fragmentations observed after the 156 amu neutral loss were similar to that of vicenin. Consequently compound **12** was concluded to be an acylated vicenin derivative.

**Table 2:** Chromatographic, spectral data and identification of acylated flavonoid glycosides in fenugreek crude seeds.

Peak	*R*_t_ (min)	λ_max_ (nm)	[M − H]^−^	Fragment Signals (*m*/*z*)	Compound Identification
10	32.28	234, 334	771	593, 503, 473, 383, 353	apigenin 6- *C*-glucosyl 8-*C*-(2''-*O*-dihydroferuloyl)-glucoside
12	34.51	232, 270, 336	749	593, 503, 473, 383, 353	vicenin derivative
15	39.58	234, 270, 348	895	563, 447, 357, 327, 284	luteolin 7- *O*-[6''-dihydrogalloyl]-glucosyl-8-*C*-pentosyl-(1→6)-glucoside
16	40.40	270, 346	895	563, 447, 357, 339, 327, 285	luteolin 7-O-[6''-dihydrogalloyl]-glucosyl-8- *C*-pentosyl-(1→2)-glucoside
18	44.22	232, 268, 336	863	563, 443, 323, 311	apigenin 7- *O*-(2''-dihydrogalloyl)-rhamonsyl-6-*C*-(2'''-pentosy)-glucoside
20	47.94	234, 270, 336	863	563, 443, 323, 311, 283	apigenin 7- *O*-(2''-dihydrogalloyl)-rhamonsyl-6-*C*-(2'''-pentosy)-glucoside
21	49.62	232, 270, 336	725	533, 443, 413, 383, 353	apigenin 6- *C*-pentosyl 8-*C*-(2''-*O*-hydroxyferuloyl)-pentoside
22	50.69	232, 272, 340	759	593, 473, 383, 353	apigenin 6- *C*-glucosyl 8-*C*-(6''-*O*-methoxygalloyl)-glucoside
23	51.74	234, 270, 336	863	563, 431, 323, 283	apigenin and 7- *O*-(6''-dihydrogalloyl)-rhamonsyl-6-*C*-(2'''-pentosy)-glucoside
24	53.33	232, 316	877	563, 473, 447, 327, 285	kaempferol 7- *O*-(6''-galloyl)-glucosyl 6-*C*-(2'''pentosyl)-rhamnoside
25	55.50	232, 270, 346	877	533, 447, 357, 339, 305, 285	luteolin 7- *O*-(2''-galloyl)-glucosyl 6-*C*-(2'''pentosyl)-rhamnoside
26	58.02	232, 270, 338	893	577, 473, 383, 353	apigenin 7- *O*-(6''-dihydrogalloyl)-glucosyl-8-*C*-rhamnosyl-6-*C*-glucoside
27	60.02	230, 270, 338	893	577, 473, 383, 353	apigenin 7- *O*-(2''-dihydrogalloyl)-glucosyl-8-*C*-rhamnosyl-6-*C*-glucoside
28	64.59	232, 270, 344	925	605, 563, 443, 383, 353	luteolin 7- *O*-(6''-quinoyl)-rhamnosyl-6-*C*-pentosyl-8-*C*,*O*-(6'''acetyl)-glucoside
29	65.60	232, 270, 344	547	487, 457, 383, 353, 283	luteolin 8- *C*-(2''-malonyl)-glucoside
30	68.87	270, 344	935	651, 547, 461, 327, 285	luteolin 7- *O*-(2''dihydrogalloyl)-pentosyl-4'-*O*-(2''',6'''-malonyl-pentosyl)-rhamnoside
32	93.20	232, 270, 318	1133	1063, 917, 577, 164, 293	kaempferol 7- *O*-(2''',6''',2''-malonyl)-rhamonsyl-diglucosyl-3-*O*-(6'''''rhamnosyl)-rhamnoside

Compound **21** presents a molecular ion at *m*/*z*: 725 [M − H]^−^. The characteristic fragment signals at *m*/*z*: 383 (Ag + 113) and *m*/*z*: 353 (Ag + 83) (−^0,3'''^X_0_), indicating a di *C*-glycosylflavone linkage were observed [48]. The fragment signals observed at *m*/*z*: 533 [(M − H) − 192]^−^ (acyl − 18), *m*/*z*: 443 [(M − H) − 192 − 90]^−^ (−^0.2''^X_0_) and *m*/*z*: 413 [(M − H) − 192 − 120]^−^ (−^0,1''^X_0_), corresponding to the loss of the an *O*-(acyl)-*C*-pentosyl with a 1→2 interglycosidic linkage due to the high relative intensity (70%) of the signal observed at *m*/*z*: 533 [50]. The observed neutral loss of a 192 amu corresponded to a dehydrated hydroxyferulic acid moiety. On the basis of these results and on the fact that the relative intensity of the ion signal at *m*/*z*: 353 (−^0,3'''^X_0_) was higher than that observed at *m*/*z*: 413 (−^0,1''^X_0_), compound **21** was proposed to be apigenin 6-*C*-pentosyl 8-*C*-(2''-*O*-hydroxyferuloyl)-pentoside.

Compound **22** presents a molecular ion at *m*/*z*: 759 [M − H]^−^. The MS data showed fragments signals at *m*/*z*: 593 [(M − H) − 166]^−^ (acyl − 18) indicating a loss of an *O*-acyl moiety. The latter was concluded to be linked through a *C*-glycosylation, due to the presence of the fragments ions *m*/*z*: 383 (Ag + 113) and *m*/*z*: 353 (Ag + 83) which is indicative of di *C*-glycosylflavone [50]. Other fragment signals were observed at *m*/*z*: 473 [(M − H) − 120]^−^ (−^0,2''^X_0_), *m*/*z*: 383 [(M − H) − 120 − 90] (−^0,3'''^X_0_) and *m*/*z*: 353 [(M − H) – 120 − 120]^−^ (−^0,2'''^X_0_) (base peak), corresponding to the loss of the diglucosyl residue [35]. On the basis of these results and on the fact that the fragment signal observed at *m*/*z*: 593 [(M − H) − 166]^−^ (acyl − 18), showed a very low relative intensity (10%), compound **22** was concluded to be apigenin 6-*C*-glucosyl 8-*C*-(6''-*O*-methoxygalloyl)-glucoside.

Compounds **15** and **16** present a molecular ion at *m*/*z*: 895 [M − H]^−^. The MS data showed fragment signals at *m*/*z*: 563 [(M − H) − (acyl − 2H) − 162]^−^, indicating an *O*-acylglycosylation on a phenolic hydroxyl through 1→6 interglycosidic linkage, due to their low relative intensity. Other fragment signals were observed at *m*/*z*: 447 [(M − H) − (acyl + 2H) − 162 − (132 − 16)]^−^, *m*/*z*: 357 [(M − H) − (acyl + 2H) − 162 − (132 − 16) − 90]^−^ (−^0,3'''^X_0_) (Ag + 71) and *m*/*z*: 327 [(M − H) − (acyl + 2H) + 162 − (132 − 16) − 120]^−^ (−^0,2'''^X_0_)(Ag + 41), corresponding to the loss of a pentosylglucoside with an interglycosidic 1→6 linkage in compound **15**, and 1→2 for compound **16**, due to a high relative intensity of the fragment ion *m*/*z*: 447 (compound **16**) than in compound **15**. The last fragment signals observed at *m*/*z*: 357 (Ag + 71) and *m*/*z*: 327 (Ag + 41) are typical of mono-glycosylflavones [31,48]. The higher intensity of the signal (Ag + 41) which was observed as base peak confirmed the 8-*C* substitution [26]. Therefore luteolin 7-*O*-[6''-dihydrogalloyl]-glucosyl-8-*C*-pentosyl-(1→6)-glucoside was attributed to compound **15** and luteolin 7-*O*-[6''-dihydrogalloyl]-glucosyl-8-*C*-pentosyl-(1→2)-glucoside was attributed to compound **16**.

Compounds **18**, **20** and **23** present a molecular ion at *m*/*z*: 863 [M − H]^−^. The MS data showed a fragment signal at *m*/*z*: 563 [(M − H) − (acyl − 18) − 146]^−^ (Y_0_^7−^), corresponding to the loss of an *O*-acylated rhamnosyl moiety. Other fragment signals were observed at *m*/*z*: 437 [(M − H) − 300 − 90]^−^ (−^0,2''''^X_0_), *m*/*z*: 443 [(M − H) − 300 − 120]^−^ (−^0,1''''^X_0_), *m*/*z*: 353 [(M − H) − 210]^−^ (−^0,3'''^X_0_), *m*/*z*: 323 [(M − H) − 300 − 240]^−^ (−^0,1''''^X_0_^0.2'''^X_0_) and *m*/*z*: 311 [(M − H) − 300 − 240 − 41]^−^ (Ag + 41). These fragmentations are in agreement with the presence of a disaccharide moiety consisting of a pentosyl and a glucosyl residues. These were concluded to be linked through an interglycosidic linkage, due to the presence of the signal at *m*/*z*: 311 (Ag + 41), which is typical of the mono *C*-glycosylflavones [26,48]. This was also confirmed through the neutral loss of a 240 (120 + 120) amu confirming this hypothesis. The interglycosidic linkage of the pentosyl glucosyl was at 2''' position with a 6 *C*-glycosylation in compounds **18** and **23**. Compound **20** presents the same fragments as compounds **18** and **23** with the fragment signal at *m*/*z*: 283 (Ag + 13) as base peak. Consequently apigenin 7-*O*-(2''-dihydrogalloyl)-rhamonsyl-6-*C*-(2'''-pentosy)-glucoside were proposed for compounds **18** and **20** and apigenin and 7-*O*-(6''-dihydrogalloyl)-rhamonsyl-6-*C*-(2'''-pentosy)-glucoside was proposed for compound **23**.

Compound **24 **and **25** present a molecular ion at *m*/*z*: 877 [M − H]^−^. The MS data showed fragment ions at *m*/*z*: 563 [(M − H) − (acyl − 18) − 162]^−^ (Y_0_^7−^), with a very low relative intensity (7%), indicating an *O*-(acyl)-glycosylation on the phenolic hydroxyl by interglycosidic 1→6 (**24**) and 1→2 linkage (**25**). Other fragment signals were observed at *m*/*z*: 473 [(M − H) – 162 − (acyl − 18) − 90]^−^ (−^0,2''''^X_0_), *m*/*z*: 447 [(M − H) − 314 − (132 − 16)]^−^ and *m*/*z*: 327 [(M − H) − 314 − (132 − 16) − 120]^−^ (−^1.5'''^X_0_). The latter’s (−^0,2''''^X_0_) and (−^1,5'''^X_0_) corresponding to the loss of the pentosyl-rhamnoside residue by interglycosidic linkage at 2''' and 6''' position [35]. The presence of the fragment ion *m*/*z*: 327 (Ag + 41), is characteristic of the mono-luteolin *C*-glycosyl derivative [48]. In compound **25**, the presence of the fragment ions at *m*/*z*: 357 (Ag + 71), *m*/*z*: 339 (Ag + 71 − 18) and *m*/*z*: 305 (Ag + 19) (base peak), more abundant in the 6-*C* than in 8-*C* isomers were observed [31,48]. These results, suggested that compounds **24** and **25** could be kaempferol 7-*O*-(6''-galloyl)-glucosyl 6-*C*-(2'''-pentosyl)-rhamnoside (**24**) and luteolin 7-*O*-(2''-galloyl)-glucosyl 6-*C*-(2'''-pentosyl)-rhamnoside (**25**).

Compounds **26** and **27** present a molecular ion at *m*/*z*: 893 [M − H]^−^. The MS data also showed fragment signals at *m*/*z*: 577 [(M − H) − (acyl − 18) − 162]^−^ (−Y_0_^7^) in agreement with the loss of an *O*-(acyl)-glucosyl (154 + 162 amu) moiety. This residue was concluded to be linked to the phenolic hydroxyl by interglycosidic 1→2 linkage (**27**), due to the high relative intensity of the signal located at *m*/*z*: 577 [51]. Other fragment signals were observed at *m*/*z*: 473 [(M − H) − 316 − 104]^−^ (−^0,2'''^X_0_), *m*/*z*: 383 [(M − H) − 316 − 104 − 90]**^−^** (−^0,3''''^X) and *m*/*z*: 353 [(M − H) − 316 − 104 − 120]^−^ (−^0,2''''^X_0_) corresponding to the loss of the rhamonsyl (−^0,2'''^X_0_) and glucosyl (−^0,2''''^X_0_) residues. The fragment signals at *m*/*z*: 383 (Ag + 113) and *m*/*z*: 353 (Ag + 83) which are characteristics of di *C*-glycosilflavone were also observed. The signal located at *m*/*z*: 353 was observed with a higher intensity in agreement with a 6-*C*-glucosyl-8-*C*-rhamnosyl-substitution. On the basis of the obtained results, these compounds were concluded to be apigenin 7-*O*-(6''-dihydrogalloyl)-glucosyl-8-*C*-rhamnosyl-6-*C*-glucoside (**26**) and apigenin 7-*O*-(2''-dihydrogalloyl)-glucosyl-8-*C*-rhamnosyl-6-*C*-glucoside (**27**).

Compound **28** presents a molecular ion at *m*/*z*: 925 [M − H]^−^. The MS showed fragment signals at *m*/*z*: 605 [(M − H) − (acyl − 18) − 146]^−^ (–Y_0_^7^), with a low relative intensity (6%), corresponding to the loss an *O*-(acyl)-rhamonsyl (174 + 146 amu) with a 1→6 interglycosidic linkage [51]. Other fragment signals were observed at *m*/*z*: 563 [(M − H) − 320 − (acyl − 18)]^−^, *m*/*z*: 443 [(M − H) − 320 − (acyl − 18) − 120]^−^ (–^0,2'''^X_0_), indicating to the Z_j_^−^ (acyl − 18) loss connected by interglycosidic linkage on the sugar moiety with a *C*-glycosylation. The ion *m*/*z*: 383 [(M − H) − 320 − (acyl − 18) − 120 − 60]^−^ (–^0,3''''^X_0_) and *m*/*z*: 353 [(M − H) − 320 − acyl − 120 − 90]^−^ (–^0,2''''^X_0_), corresponding to the loss of the glucosyl and pentosyl residue [35,45] were also observed. The fragment signals at *m*/*z*: 383 (Ag + 113) and *m*/*z*: 353 (Ag + 83) typical of the di *C*-glycosylflavone were also observed. The relative intensity of the [(M − H) − 320 − (acyl − 18) − 120 − 90]^−^ signal was higher than that of [(M − H) − 320 − (acyl − 18) − 120]^−^ in agreement with a 6-*C*-pentosyl-8-*C*-glucosyl- substitution. These results suggested that compound **28** was luteolin 7-*O*-(6''-quinoyl)-rhamnosyl-6-*C*-pentosyl-8-*C*,*O*-(6'''-acetyl)-glucoside.

Compound **29** presents a molecular ion at *m*/*z*: 547 [M − H]^−^. The MS data showed fragment ion signals at *m*/*z*: 457 [(M − H) − 90]^−^ (–^0,3''^X_0_), *m*/*z*: 353 [(M − H) − 90 − 104]^−^ and *m*/*z*: 327 [(M − H) − 116 − 104]^−^ (Ag + 41), *m*/*z*: 297 [(M − H) − 146 − 104]^−^ and *m*/*z*: 283 [(M − H) − 164 − 86 − 14]^−^. The neutral loss (–^0,3''^X_0_) and 104 amu was concluded to correspond to the loss of the *O*-(malonyl)-*C*-glucosyl by interglycosidic 1→2 linkage, due to a high relative intensity of the ion *m*/*z*: 353. Moreover, the fragment ion signal observed at *m*/*z*: 327 (Ag + 41), is typical of mono *C*-glycosylflavones and confirmed the substitution in the *C*-8 position due to the low relative intensity of the fragment ion *m*/*z*: 327 [26]. These results suggested that compound **29** could be luteolin 8-*C*-(2''-malonyl)-glucoside.

Compound **30** presents a molecular ion at *m*/*z*: 935 [M − H]^−^. The MS data also showed a fragment signal at *m*/*z*: 651 [(M − H) − (acyl − 18 − 2H) − 132]^−^ (Y_0_^7^) corresponding to the loss of the an *O*-(acyl)-pentosyl with a 1→2 interglycosidic linkage due to its very high relative intensity (81%). Other fragment signals were observed at *m*/*z*: 547 [(M − H) − 284 − 104]^−^ (^1,''''^X_0_), *m*/*z*: 461 [(M − H) − 284 − 104 − 86]^−^, *m*/*z*: 327 [(M − H) − 284 − 190]^−^ (^0,1'''^X_0_) (Ag + 41) and *m*/*z*: 285 [Ag − H]^−^ corresponding to the loss of the *O*-(malonyl)-pentosyl-rhamnosyl- unit with 1→2 and 1→6 interglycosidic linkages due to the presence the fragment signal *m*/*z*: 285 (aglycone) [31]. On the basis of these results compound **30** was concluded to be luteolin 7-*O*-(2''-dihydrogalloyl)-pentosyl-4'-*O*-(2''',6'''-malonyl-pentosyl)-rhamnoside.

Compound **32** presents a molecular ion at *m*/*z*: 1133 [M − H]^−^. Other fragment signals were observed at *m*/*z*: 1063 [(M − H) − 70]^−^ (base peak), *m*/*z*: 917 [(M − H) – 70 − 146]^−^ (Y^7^_6'''_), *m*/*z*: 577 [(M − H) − 86 − 146 − 324]^−^ (−Y^7^_2'''_Y^7^_0_) corresponding to the loss an *O*-(malonyl)-rhamnosyl-diglucosyl with 1→2 and 1→6 interglycosidic linkages according to relative intensity of the fragments ions. Additional fragment signals were observed at *m*/*z*: 413 [(M − H) – 556 − 164]^−^ (−Z^3^_6'''''_) and *m*/*z*: 293 [(M − H) − 556 − 164 − 120]^−^ (−^1,5'''''^X_0_^3^), suggesting another loss of the *O*-dirhamnosyl moiety. The relative intensity of the ion signal at *m*/*z*: 293 was higher than that of *m*/*z*: 413 indicating a 3-*O*-dirhamnosyl-aglycone. These results suggested this compound could be kaempferol 7-*O*-(2''',6''',2''-malonyl)-rhamonsyl-diglucosyl-3-*O*-(6'''''-rhamnosyl)-rhamnoside.

### 2.3. Hydroxycinnamic Acids

Eight hydroxycinnamic acid derivatives with low concentrations were detected in the studied fenugreek seeds sample. The tentative characterization of these compounds was based on UV spectra, mass spectral data and comparision with the literature data [52,53]. Different derivatives of hydroxycinnamic acids (caffeic acid, dihydrogallic acid, sinapic acid, gallic acid, coumaric acid) were thus detected in the studied sample with a predominance of caffeic acid derivatives.

Compound **1** presents a molecular ion at *m*/*z*: 827 [M − H]^−^ (Table 3). The MS data showed fragment signals at *m*/*z*: 665 [(M − H) − 162]^−^, *m*/*z*: 503 [(M − H) − 324], *m*/*z*: 383 [(M − H) − 324 − 120]^−^, *m*/*z*: 341 [(M − H) − 324 − 162]^−^ , *m*/*z*: 281 [(M − H) − 324 − 162 − 60]^−^, *m*/*z*: 221 [(M − H) − 324 − 162 − 120]^−^, and *m*/*z*: 179 [(M − H) − 324 − 162 − 162]^−^. The loss of 324 amu is in agreement with the presence of a diglucoside moiety [54]. The last fragment corresponded to the loss of a caffeic acid moiety. These results indicated that this compound could be tricaffeoyl-glucosyl-glucoside.

Compound **2** presents a molecular ion at *m*/*z*: 695 [M − H]^−^. The MS data showed fragment signals at *m*/*z*: 619 [(M − H) − 76]^−^, with a high relative intensity (90%), *m*/*z*: 407 [(M − H) − 76 − 212], with a low relative intensity (6%), *m*/*z*: 309 [(M − H) − 386]^−^ (base peak) and *m*/*z*: 180 [(M − H) − 515]^−^ with a relative intensity of 66%. The last fragment signal indicated the loss of the caffeic acid molecule while the 515 amu indicated the loss of the deshydrated dicaffeoyl-hydroxyferulic acid. Therefore, this compound was tentatively identified as tricaffeoyl-hydroxyferulic acid.

Compound **4** presents a molecular ion at *m*/*z*: 947 [M − H]^−^. The MS data showed fragment signals at *m*/*z*: 765 [(M − H) − 182]^−^ (loss of hydrocaffeic acid), *m*/*z*: 382 [(M − H) − 182 − 383]^−^ (loss of dehydrated hydro feruloyl-sinapic acid), *m*/*z*: 205 [(M − H) − 182 − 383 − 177]^−^ (loss of sinapic acid). These results, suggested that this compound could be disynapoyl-hydro feruloyl-feruloyl-hydrocaffeic acid.

Compound **5** presents a molecular ion at *m*/*z*: 447 [M − H]. Other signals were observed at *m*/*z*: 224 [(M − H) − 223]^−^, *m*/*z*: 152 [(M − H) − 295]^−^. The loss of the 295 amu is in agreement with a coumaroyl-pentosyl-unit. The last fragment ions corresponded to the loss of the dehydrated gallic acid unit, suggesting that this compound could be galloyl-coumaric acid pentoside.

Compound **6** presents a molecular ion at m/z 499 [M − H]^−^. The MS data showed fragment signals at *m*/*z*: 377 [(M − H) − 122]^−^, *m*/*z*: 273 (M − H) − 226]^−^, *m*/*z*: 163 [(M − H) − 336]^−^ and *m*/*z*: 119 [(M − H) − 336 − 44]^−^. The loss of the 336 amu, indicated to the loss of a dehydrated caffeoyl-quinic acid unit and the last fragment ion observed at *m*/*z*: 163, corresponded to the loss of a coumaroyl radical, suggesting that this compound could be a caffeoyl-coumaroyl-quinic acid.

Compound **7** presents a molecular ion at *m*/*z*: 801 [M − H]^−^. The MS data also showed fragment signals at *m*/*z*: 671 [(M − H) − 130]^−^, *m*/*z*: 477 [(M − H) − 324]^−^ corresponding to the loss of diglucosyl. Other signals were observed at *m*/*z*: 323 [(M − H) − 324 − 154]^−^ (loss of protocatechuic acid), *m*/*z*: 144 [(M − H) − 324 − 154 − 179]^−^ (loss of caffeic acid radical). These results suggested that this compound could be dicaffeoyl-protocatechuic acid diglucoside.

### 2.4. Quantitation of Flavonoids Glycoside in Crude Fenugreek Seeds

After having studied the qualitative phytochemical composition of the crude fenugreek seeds extract, the quantitative analysis of the individual identified phenolic compounds was explored and the obtained results are gathered in Table 4. The quantitation of the detected compounds have been made on the basis of the area % calculation procedure which reports the area of each peak in the chromatogram as a percentage of the total area of all peaks. This method supposes that all components respond equally in the detector and are all eluted from the column. Even if these criteria are may be not assured, the used method provides at least a suitable approximation of the relative amounts of the detected compounds.

The obtained results showed that the identified compounds are present at different percentages, with compound **16**, an acylated triglycoside derivative of luteolin, as a major compound (15.80%). This was followed by compounds **14** (14.41%) and **20** (12.02%), which are glycosides of apigenin. The non-acylated apigenin (**17** and **11**) and kaempferol (**31**) derivatives were present with similar percentages (8.82%, 9.61% and 8.71% respectively). The acylated apigenin glycosides (**18**) and luteolin (**13**) compounds were present with 6.59% and 5.60% respectively. Finally compounds **15** and **32**, which are acylated derivatives of luteolin and kaempferol were present at 3.76% and 2.52% respectively. These flavonoids represent the major compounds with 87.80% of the total identified compounds. The other compounds were present at lower relative percentages.

**Table 3:** Chromatographic, spectral data and identification of phenolic acids in fenugreek crude seeds.

Peak	*R*_t_ (min)	λ_max_ (nm)	[M − H]^−^	Fragment Signals (*m*/*z*)	Compound Identification
1	9.23	228, 264	827	665, 545, 383, 341, 281, 221, 179, 146, 129, 110	tricaffeoyl-glucosyl-glucoside
2	13.09	232, 276	695	619, 407, 363, 309,180, 167,128	tricaffeoyl-hydroxyferulic acid
3	17.65	248	888	863, 452, 431, 171, 137	dihydrogallic acid derivative
4	19.22	228, 260	947	765, 483, 382, 266, 205, 167, 115	disynapoyl-hydro feruloyl-feruloyl-hydrocaffeic acid
5	20.28	236, 278, 328	447	224, 152, 136, 108	galloyl-coumaric acid pentoside
6	21.40	230, 296	499	377, 273, 163, 119	caffeoyl-coumaroyl-quinic acid
7	23.38	240, 334, 346	801	671, 477, 399, 323, 261, 144, 119	dicaffeoyl-protocatechuic acid diglucoside
8	26.46	220, 234, 316	837	647, 625, 587, 452, 395, 347, 317, 293, 165, 132, 128, 115	unidentified

**Table 4:** Quantitative chemical composition of the fenugreek crude seeds (results are given in %).

Peak Number	Quantitative Chemical Composition (%)	Peak Number	Quantitative Chemical Composition (%)	Peak Number	Quantitative Chemical Composition (%)
1	0.11	12	0.66	23	0.26
2	0.15	13	5.60	24	0.80
3	3.10	14	14.41	25	0.08
4	0.45	15	3.76	26	0.05
5	0.43	16	15.80	27	0.38
6	0.27	17	8.82	28	0.45
7	0.34	18	6.59	29	0.44
8	0.70	19	1.18	30	0.62
9	0.27	20	12.02	31	8.71
10	0.82	21	0.31	32	2.51
11	9.61	22	0.31		

The quantitative composition of the studied crude seeds was dominated by non-acylated flavonoid glycosides (48.60%), followed by acylated flavonoid glycosides (45.86%) and finally phenolic acids (5.55%). Apigenin was the major constitutive aglycon (61.30%), followed by luteolin (21.15%) and kaempferol (11.22%). This showed that the studied sample was dominated by flavone derivatives (83.20%).

The data obtained thus demonstrates that acylated and non-acylated flavone derivatives, with apigenin as the main aglycon, dominate the phenolic composition of crude fenugreek seeds.

## 3. Materials and Methods

### 3.1. General

All the chemicals used were of analytical grade and were obtained from Sharlab S.L. (Barcelona, Spain). Apigenin, luteolin, kaempferol, apigenin 8-*C*-neohesperidoside, luteolin 7-*O*-glucoside, kaempferol 7-*O*-neohesperidoside and apigenin 7-*O*-neohesperidoside were obtained from Extrasynthese (Genay Cedex, France).

### 3.2. Plant Materials

Crude *Trigonella foenum-graecum* seeds were collected in June 2009 from the north Morocco area. Dried seed samples were ground to a powder using a commercial coffee grinder (Taurus, Barcelona, Spain) and stored in plastic bags and kept in the dark at room temperature until extraction.

### 3.3. Accelerated Solvent Extractor (ASE)

Extraction was carried out using a Dionex ASE 200 Accelerated Solvent Extractor. This technique is also known as pressurized liquid extraction or pressurized fluid extraction. The system, automated and capable of sequential extractions, was made up of stainless steel extraction cells and its programmed parameters (temperature and pressure) were kept at their specified values by electronically controlled heaters and pumps. In this work, the extraction conditions were based on US EPA method [55]. The used solvents were hexane for defatting and a mixture of methanol/water (50/50, *v*/*v*) for polyphenols extraction. The temperature was 80 °C and the pressure was 1500 psi (10.3 Mpa) with 5 min for heating, 8 min as static time and 3 cycles for extraction. In the end, the extraction cell was flushed with solvent (60% cell volume) and purged with nitrogen (120 s). Three replicate extractions for each experimental condition were performed (*n* = 3). The extraction procedure was as follows: (1) Sample is loaded in the cell, (2) Cell is filled with solvent up to a pressure of 10.34 MPa, (3) Initial heat-up time is applied, (4) A static extraction with all system valves closed is performed, (5) The cell is rinsed (with 60% cell volume using extraction solvent), (6) Solvent is purged from the cell with N_2_ gas and (7) Depressurization takes place.

At the end, the extracts were dried under a nitrogen flow at 40 °C, using Turbovap LV Concentration Evaporator (Caliper, Lifescience, Hopkinton, MA, USA). The residue was redissolved in 2 mL of methanol/water (50/50, *v*/*v*) mixture and filtered through a 0.45 µm PVDF syringe filter, and analyzed by HPLC.

### 3.4. HPLC–DAD–ESI/MS Analyses

The HPLC–DAD–ESI/MS apparatus used consisted of a Waters 717 plus autosampler HPLC coupled to a Diode array detector and mass spectrometer (Waters 600 controllers, Hewlett-Packard series 1100 MSD, Agilent Technologies, Aldbronn, Germany). The separation was performed on a reversed-phase Waters Nova-Pak C_18_ (4.9 × 250 mm, 4 µm particle size) column (Water Milford, MA, USA) at room temperature. The mobile phase consisted of water/AcOH (98/2) (solvent A) and acetronitrile/AcOH (98/2) (solvent B). Separation was performed using the following elution gradient: 0 to 80% B linear during 55 min, from 80% to 90% B during 2 min, 90% B isocratic during 13 min, from 90% to 95% B during 10 min and from 95% to 100% B during 10 min. This was followed by a washing with methanol and reequilibration of the column from 90 to 120 min. The flow rate was 1 mL/min, and the injection volume was 10 µL. The analysis was performed at 280, 310, 330 and 360 nm. Mass spectra were acquired using electrospray ionization in the negative ion mode scanning from *m*/*z*: 100 to 3000 using the following fragmentation program: from *m*/*z*: 0 to 200 (100 V) and from *m*/*z*: 200 to 3000 (200 V). Ionization parameters were as follows: drying gas (N_2_) at a flow of 10 L/min and a temperature of 350 °C. The nebulizer pressure was 55 psi and the capillary voltage was 4000 V.

## 4. Conclusions

In this study, HPLC coupled to both ESI/MS and DAD was used to separate and identify 24 flavonoid glycosides in the extract of fenugreek crude seeds. Some minor phenolic acids were also detected and characterized. Both acylated and non-acylated flavonoid derivatives were detected. Most of the identified flavones were apigenin adducts followed by luteolin derivatives, while only two kaempferol glycosides were detected as flavonols. This work presents thus a more complete description of the phenolic compounds present in the crude fenugreek seeds. The obtained results indicate that crude fenugreek seeds could be considered as a rich source of bioactive phenolic compounds. Due to the widely reported antioxidant activity of flavonoids, crude fenugreek seeds could be exploited as an important supplement in food manufacturing such as functional foods or other herbal preparations.
